# Safety of cilostazol in peripheral artery disease: a cohort from a primary healthcare electronic database

**DOI:** 10.1186/s12872-018-0822-4

**Published:** 2018-05-08

**Authors:** Jordi Real, M Catalina Serna, Maria Giner-Soriano, Rosa Forés, Guillem Pera, Esther Ribes, Maite Alzamora, Josep Ramon Marsal, Antonio Heras, Rosa Morros

**Affiliations:** 1 Unitat de Suport a la Recerca Barcelona, Institut Universitari d’Investigació en Atenció Primària Jordi Gol (IDIAPJGol), Barcelona, Spain; 2 Epidemiologia i Salut Pública, Universitat Internacional de Catalunya, Sant Cugat, Spain; 3 CIBER de Diabetes y Enfermedades Metabólicas Asociadas (CIBERDEM), Instituto de Salud Carlos III (ISCIII), Madrid, Spain; 4 Unitat de Suport a la Recerca Lleida, Institut Universitari d’Investigació en Atenció Primària Jordi Gol (IDIAPJGol), Lleida, Spain; 5 Universitat de Lleida, Lleida, Spain; 6 Institut Català de la Salut, Lleida, Spain; 7 Institut Universitari d’Investigació en Atenció Primària Jordi Gol (IDIAPJGol), Gran Via de les Corts Catalanes 587, àtic, 08007 Barcelona, Spain; 8 Universitat Autònoma de Barcelona, Bellaterra (Cerdanyola del Vallès), Gran Via de les Corts Catalanes 587, àtic, 08007 Barcelona, Spain; 9 Institut Català de la Salut, Gran Via de les Corts Catalanes 587, àtic, 08007 Barcelona, Spain; 10 Centre d’Atenció Primària Riu Nord-Riu Sud Santa Coloma de Gramenet. Direcció d’Atenció Primària Metropolitana Nord. Institut Català de la Salut, Barcelona, Spain; 11 Unitat de Suport a la Recerca Metropolitana Nord, Institut Universitari d’Investigació en Atenció Primària Jordi Gol (IDIAPJGol), Mataró, Spain; 12 Unitat de Farmàcia. Direcció d’Atenció Primària Lleida. Institut Català de la Salut, Lleida, Spain; 13 Epidemiology Unit of the Cardiovascular Service, Hospital Universitari Vall d’Hebron, CIBER of Epidemiology and Public Health (CIBERESP), Instituto de Salud Carlos III Barcelona, Barcelona, Spain; 14 Plataforma SCReN (Spanish Clinical Research Network), Unidad de Investigación Clínica y Ensayos Clínicos (UICEC) IDIAPJGol, Barcelona, Spain

**Keywords:** Cilostazol, Pentoxifylline, Peripheral artery disease, Primary healthcare, Electronic health records, Haemorrhage, Arrhythmia, Cardiovascular events, Coronary artery disease

## Abstract

**Background:**

Cilostazol has been associated with spontaneous reports of cardiovascular adverse events and serious bleeding. The objective of this study is to determine the relative risk of cardiovascular adverse events or haemorrhages in patients with peripheral artery disease treated with cilostazol in comparison to pentoxifylline users.

**Methods:**

Population-based cohort study including all individuals older than 40 who initiated cilostazol or pentoxifylline during 2009–2011 in SIDIAP database. The two treatment groups were matched through propensity score (PS).

**Results:**

Nine thousand one hundred twenty-nine patients met inclusion criteria and after PS matching, there were 2905 patients in each group. 76% of patients were men, with similar mean ages in both groups (68.8 for cilostazol and 69.4 for pentoxifylline). There were no differences in bleeding, cerebrovascular and cardiovascular events between both groups.

**Conclusions:**

Patients treated with cilostazol were different from those treated with pentoxifylline at baseline, so they were matched through PS. We did not find differences between treatment groups in the incidence of bleeding or cardiovascular and cerebrovascular events. Cilostazol should be used with precaution in elderly polymedicated patients.

**Electronic supplementary material:**

The online version of this article (10.1186/s12872-018-0822-4) contains supplementary material, which is available to authorized users.

## Background

Peripheral artery disease (PAD) prevalence and incidence are both age-related. Its prevalence in population older than 60 ranges between 8.6 to 24.2% in women and 5.5 to 24.7% in men [1, 2] and it increases progressively with ageing population [1]. Population studies conducted in Spain have found a prevalence of PAD between 3.7 and 7.6% in general population [3, 4]. PAD causes an impairment in quality of life, decrease in life expectancy and it is an important predictor of morbidity and mortality [5, 6].

Many people with PAD do not have symptoms, but when they occur, intermittent claudication (IC) is the main symptom. Treatment for IC is a combination of preventive measures such as modification of risk factors, physical activity, treatment of symptoms and antiplatelet therapy [7, 8]. Currently, there are two drugs authorized for PAD treatment; pentoxifylline [9] and cilostazol [10]. Cilostazol was approved by European Medicines Agency (EMA) in 2002 and in Spain in 2009 to improve walking distances in patients with IC [10].

Cilostazol is a phosphodiesterase III inhibitor which was first approved for the treatment of symptoms related to IC. It is a potent platelet-aggregation inhibitor and has arterial vasodilatory effects [11]. Cilostazol is contraindicated in patients with severe renal impairment, moderate or severe hepatic impairment, and known predisposition to bleeding and in patients with history of ventricular tachycardia, ventricular fibrillation or multifocal ventricular ectopic beats, or prolongation of the QTc interval. Cilostazol has been associated with a number of spontaneous reports of cardiovascular adverse effects (myocardial infarction, angina, and arrhythmias) and serious bleeding [10, 12]. Haemorrhagic events in elderly patients co-treated with antiplatelets were also reported by the Centro Autonómico de Farmacovigilancia de Cantabria [13].

The EMA evaluated the benefit/risk of cilostazol in a referral and recommended changes in the summary of product characteristics (SmPC), including extension of contraindications to patients with unstable angina pectoris, recent acute myocardial infarction (AMI), or recent coronary intervention. EMA also highlighted cautions and concerns over haemorrhagic and vascular events [12, 14]. Healthcare professionals in Europe have been advised to use cilostazol for IC only in patients where other lifestyle modifications such as smoking cessation and exercise have not provided adequate improvement and to continue use only in those patients who have shown clinically relevant benefit after 3 months of therapy. Other cautions include avoiding cilostazol in patients receiving two or more additional antiplatelet or anticoagulant agents and potentially avoiding use or decreasing the dose in patients concomitantly receiving strong inhibitors of CYP3A4 or CYP2C19 [12].

Cilostazol is recommended by guidelines as an effective therapy in improving symptoms and increasing walking distance in patients with lower extremity PAD [15, 16]. Nevertheless, the effect of cilostazol on morbidity and mortality has not been fully determined. In this population-based primary healthcare (PHC) cohort study, we assess the safety of cilostazol for the treatment of PAD patients in terms of incidence of cardiovascular events, arrhythmias and haemorrhages during the follow-up.

The main objective of the study was to calculate the relative risk of cardiovascular adverse events or bleeding in patients with PAD treated with cilostazol in comparison to pentoxifylline users. The specific objectives were: 1) to calculate risks for cilostazol users versus pentoxifylline users of: ischemic or coronary artery disease (CAD), arrhythmias or haemorrhages; 2) to determine whether the coexistence of type 2 diabetes mellitus (DM) may increase adverse events of cilostazol; and 3) to determine whether concomitant use of antiplatelet agents with cilostazol increases the risk of bleeding.

## Methods

### Design

This is a population-based retrospective observational cohort study.

### Population

The study population were all individuals older than 40 years with a new prescription of cilostazol or pentoxifylline between 2009 and 2011, from 274 PHC teams from the Catalan Health Institute (Institut Català de la Salut, ICS), which is the main health provider in Catalonia, with a reference population of 5,835,000 patients (80% of the Catalan population).

We excluded patients with less than two visits to the PHC centre during the year before the inclusion and patients with only one dispensing of the drugs of interest during the study period.

All patients were followed-up from the cohort entry date up to 31st December 2013, death or lost to follow-up.

### Data source

The main data source is SIDIAP (Information System for Research in Primary Care), [17] which contains anonymized clinical information of all PHC centres of ICS. This information emerges from ECAP™, electronic health records in PHC of the ICS, and it includes socio-demographic characteristics, health conditions registered as ICD10 codes, clinical parameters, toxic habits, laboratory data, and General Practitioners’ prescriptions with their corresponding pharmacy invoice data. SIDIAP may be linked with CMBD-HA (“minimum set of data at hospital discharge”), [18] which contains diagnoses coded with ICD9 at hospital discharge from all hospitals in Catalonia, to obtain the data for comorbidities and for the endpoints of the study.

### Variables collected at baseline

The following variables were collected from SIDIAP database: socio-demographic characteristics, smoking status, body mass index (BMI, kg/m^2^), laboratory data (total cholesterol and LDL-cholesterol determinations, creatinine and estimated glomerular filtration calculated by MDRD), blood pressure (BP) determinations, ankle-brachial pressure index (ABPI) measures, diagnosis of PAD, and other comorbidities of interest (hypertension, type 2 DM, dyslipidemia). The following variables were extracted from SIDIAP and CMBD-HA: previous history of haemorrhages (total and specific gastrointestinal and cerebral haemorrhages), stroke, CAD, and arrhythmias.

Exposure to drugs of interest (cilostazol and pentoxifylline) and to comedications (diuretics, β-blockers, calcium channel antagonists, angiotensin converter enzyme inhibitors (ACEI), angiotensin receptor blockers (ARB), nitrates and other vasodilators, lipid-modifying agents, antidiabetic drugs and insulins, proton pump inhibitors, non-steroidal anti-inflammatory drugs (NSAID), oral anticoagulants (OAC), and antiplatelets) were obtained from the pharmacy invoice registry, which contains all information on pharmaceutical products dispensed by community pharmacies with ICS prescriptions, by ATC codes. All diagnosis codes as ICD9 and/or ICD10 and ATC drug codes may be found at (Additional file 1: Table S1).

### Outcomes of interest

We collected the following events from CMBD-HA during the study period: haemorrhages (total and specific gastrointestinal and cerebral haemorrhages), stroke, CAD, and arrhythmias.

### Sample, matching process, and statistical power

The two cohorts were matched in order to balance socio-demographic characteristics, comorbidity and comedications. The method used was the “Nearest Neighbour”, which is based on Propensity Score (PS) Link logit with “MatchIt” library from R (v3.0.1).

The variables used to build this PS were: sex, age, BMI, smoking status, comorbidities, BP control, estimated glomerular filtration as per MDRD, ABPI and co-medications at baseline.

The final matched sample included 5810 individuals, 2905 per group. After the matching process, a 36.4% of the sample (*n* = 3319) was removed and the potential bias between the two samples (overall vs matched) was reduced in an 83%. Assuming that a cohort of 5810 patients with a 5-years follow-up period had an incidence of a cardiovascular event of 4.3% in one of the groups (incidence data of symptomatic patients from ARTPER study [3]), and between 1% (HR = 1.23) to 2% (HR = 1.46) of events attributable to cilostazol, the statistical power would be 53–96%. This approximation has been carried out with a Log-Rank test with an alpha-level of 5% in a bilateral contrast.

### Statistical analysis

Descriptive statistics were used to summarize overall information. In order to compare the baseline characteristics between the treatment groups, Chi-square test was used for categorical variables and Student’s t-test for quantitative variables.

Conditional Cox regression models were used to estimate incidence rates and hazard ratios (HR) and the person/time value was used as offset. Risk functions and HR were estimated with their 95% confidence intervals (CI) to compare the two groups. 95% CI and *p*-values were calculated by robust standard errors (by clusters). Goodness of fit and proportional hazards assumption of Cox models were assessed through Schoenfeld residuals method.

These analyses were conducted in the population of 5810 patients and in three subgroups of patients: > 65 years-old, patients diagnosed with type 2 DM and those co-treated with antiplatelet agents.

All statistical tests were two-sided at the 5% significance level. The analyses were performed using SPSS-IBM PC v.18 and Stata v.11 (Stata Corp., Collage Station, TX).

## Results

During the study period, 9129 patients met the inclusion criteria; 3345 were receiving treatment with cilostazol and 5784 with pentoxifylline. Patients in the two groups were different in most baseline characteristics (Table 1).

**Table 1:** Baseline characteristics of the population included before (*n* = 9129) and after (*n* = 5810) propensity score matching: socio-demographics, comorbidities, laboratory determinations, comedications

Variables	Before PS-matching			After PS-matching		
N (%)	Cilostazol (*n* = 3345)	Pentoxifylline (*n* = 5784)	*p*-value	Cilostazol (*n* = 2905)	Pentoxifylline (*n* = 2905)	*p*-value
Gender						
Women	711(21.3)	2444 (42.3)	0.001	695 (23.9)	676 (23.3)	0.557
Men	2634 (78.7)	3340 (57.7)		2210 (76.1)	2229 (76.7)	
Mean age (SD)	68.5 (11.3)	70.1 (13.4)	0.001	68.8 (11.4)	69.4 (12.2)	0.058
PAD coded	1394 (41.7)	968 (16.7)	0.001	985 (33.9)	918 (31.6)	0.061
ABPI						
< 0.7	195 (5.8)	214 (3.7)	0.001	156 (5.4)	146 (5.0)	0.359
≥ 0.7	151 (4.5)	248 (4.3)		132 (4.5)	154 (5.3)	
Missing	2999 (89.7)	5322 (92.0)		2617 (90.1)	2605 (89.7)	
Smoking status						
Non-smoker	1037 (31.0)	2666 (46.1)	0.001	955 (32.9)	1102 (37.9)	< 0.001
Smoker	1057 (31.6)	1085 (18.8)		869 (29.9)	725 (25.0)	
Ex-smoker	944 (28.2)	1157 (20.0)		792 (27.3)	760 (26.2)	
Missing	307 (9.2)	876 (15.1)		289 (9.9)	318 (10.9)	
BMI, kg/m^2^, mean (SD)	28.3 (4.3)	28.7 (5.0)	0.017	28.4 (4.4)	28.5 (4.7)	0.505
< 25	295 (8.8)	482 (8.3)	0.001	253 (8.7)	271 (9.3)	
25–30	712 (21.3)	913 (15.8)		603 (20.8)	538 (18.5)	
> 30	438 (13.1)	789 (13.6)		382 (13.1)	419 (14.4)	
Missing	1900 (56.8)	3600 (62.2)		1667 (57.4)	1677 (57.7)	
Hypertension	2059 (61.6)	3396 (58.7)	0.008	1794 (61.8)	1811 (62.3)	0.646
Type 2 DM	1296 (38.7)	1876 (32.4)	0.001	1112 (38.3)	1120 (38.6)	0.829
Dyslipidemia	1531 (45.8)	2227 (38.5)	0.001	1271 (43.8)	1285 (44.2)	0.711
CAD	533 (15.9)	803 (13.9)	0.008	461 (15.9)	461 (15.9)	1
AF	152 (4.5)	457 (7.9)	0.001	145 (5.0)	177 (6.1)	0.067
Other arrhythmias	20 (0.6)	39 (0.7)	0.661	15 (0.5)	23 (0.8)	0.193
Prior stroke	313 (9.4)	506 (8.7)	0.327	268 (9.2)	286 (9.8)	0.421
Cerebral haemorrhage	10 (0.3)	24 (0.4)	0.381	10 (0.3)	6 (0.2)	0.317
Digestive haemorrhage	63 (1.9)	123 (2.1)	0.428	31 (1.1)	25 (0.9)	0.420
Other haemorrhages	81 (2.5)	160 (4.2)	0.323	75 (2.6)	74 (2.5)	0.934
BP						
Bad control (≥140/90 mmHg)	1117 (33.4)	1605 (27.7)	0.001	954 (32.8)	880 (30.3)	0.041
Systolic BP, mean (SD)	137.9 (15.6)	135.9 (15.3)	0.001	137.9 (15.6)	136.2 (14.9)	< 0.001
Diastolic BP, mean (SD)	74.7 (9.3)	74.5 (8.9)	0.347	74.7 (9.3)	74.5 (9.0)	0.405
Total						
cholesterol, mg/dL, mean (SD)	193.3 (42.2)	194.8 (41.5)	0.269	194.0 (42.1)	193.6 (42.8)	0.801
LDL cholesterol, mg/dL, mean (SD)	116.8 (36.4)	117.8 (35.4)	0.440	117.3 (36.4)	115.9 (35.8)	0.346
≤ 100	479 (34.3)	688 (31.8)	0.119	401 (33.9)	407 (34.4)	0.068
> 100	916 (65.7)	1474 (68.2)		783 (66.1)	777 (65.6)	
≤ 150	1135 (81.4)	1764 (81.6)	0.864	960 (81.1)	979 (82.7)	0.311
> 150	260 (18.6)	398 (18.4)		224 (18.9)	205 (17.3)	
eGFR (MDRD), mL/min/1.73m^2^						
≤ 30	30 (0.9)	83 (1.4)	0.001	27 (0.9)	31 (1.1)	0.728
30–59	387(11.6)	754 (13.0)		352 (12.1)	374 (12.9)	
≥ 60	1222 (36.5)	1854 (32.1)		1021 (35.1)	1027 (35.4)	
Missing	1706 (51.0)	3093 (53.5)		1505 (51.8)	1473 (50.7)	
Diuretics	863 (25.8)	1905 (32.9)	< 0.001	796 (27.4)	813 (28.0)	0.618
β-blockers	625 (18.7)	1082 (18.7)	0.979	560 (19.3)	546 (18.8)	0.640
Calcium channel antagonists	721 (21.6)	1216 (21.0)	0.550	639 (22.0)	635 (21.9)	0.899
ACEI*	1288 (38.5)	1931 (33.4)	0.001	1102 (37.9)	1092 (37.6)	0.787
ARB*	949 (28.4)	1444 (25.0)	0.001	833 (28.7)	857 (29.5)	0.488
Vasodilators	397 (11.9)	779 (13.5)	0.028	360 (12.4)	372 (12.8)	0.635
Lipid modifying agents	2077 (62.1)	2652 (45.9)	0.001	1716 (59.1)	1698 (58.5)	0.631
Antidiabetic drugs and insulins	1275 (38.1)	1793 (31.0)	< 0.001	1104 (38.0)	1079 (37.1)	0.498
Proton pump inhibitors	1967 (58.8)	3541 (61.2)	0.023	1724 (59.3)	1759 (60.6)	0.349
NSAID*	1233 (36.9)	2290 (39.6)	0.010	1085 (37.3)	1094 (37.7)	0.807
OAC*	342 (10.2)	696 (12.0)	0.009	301 (10.4)	311 (10.7)	0.669
Antiplatelets	2169 (64.8)	2740 (47.4)	0.001	1797 (61.9)	1776 (61.1)	0.571

* *PS* propensity score, *SD* standard deviation, *PAD* peripheral artery disease, *ABPI* ankle-brachial pressure index, *BMI* body mass index, *DM* diabetes mellitus, *CAD* coronary artery disease, *AF* atrial fibrillation, *BP* blood pressure, *eGFR* estimated glomerular filtrate rate, *MDRD* modification of diet in renal disease formula, *ACEI* angiotensin-converter enzyme inhibitors, *ARB* angiotensin-receptor blockers, *NSAID* non-steroidal anti-inflammatory drugs, *OAC* oral anticoagulants

** ABPI, mean value in the last 6 months. BP, mean values in the last year. Cholesterol measures, last value in prior 6 months. eGFR, mean value in the last 6 months. Co-medications, 6 months prior to inclusion

After PS matching, there were 2905 patients per group. As shown in Table 1, most patients (76%) in both cohorts were men. Their mean ages were similar in both groups. There were differences between groups in the frequency of smokers; more than 25% of patients in both groups were current smokers. The percentages of missing values of this variable were 9.9 and 10.9% in cilostazol and pentoxifylline users, respectively.

We analysed the frequency and incidence of bleeding, cerebrovascular and cardiovascular events comparing the two cohorts (Table 2). There were no statistically significant differences between them for the overall population included.

**Table 2:** Frequency and incidence of events in the study population (*n* = 5180)

		Risk at 5 years				
Outcomes	N	(%)	CI95%	HR	CI95%	*p* value^a^
Gastrointestinal haemorrhage						
Cilostazol	35	1.2	0.8–1.6	1.10	0.7–1.8	0.711
Pentoxifylline	31	1.1	0.7–1.4			
Cerebral haemorrhage						
Cilostazol	23	0.8	0.5–1.1	0.79	0.5–1.4	0.392
Pentoxifylline	30	1.0	0.7–1.4			
Other haemorrhages						
Cilostazol	92	3.2	2.5–3.8	0.83	0.6–1.2	0.267
Pentoxifylline	115	4.0	3.2–4.7			
Any haemorrhage						
Cilostazol	115	4.0	3.2–4.7	0.82	0.6–1.1	0.119
Pentoxifylline	143	4.9	4.1–5.7			
CAD						
Cilostazol	287	9.9	8.8–11.0	0.94	0.8–1.1	0.433
Pentoxifylline	305	10.5	9.4–11.6			
Stroke						
Cilostazol	161	5.5	4.7–6.4	0.94	0.8–1.2	0.564
Pentoxifylline	171	5.9	5.0–6.7			
AF						
Cilostazol	194	6.7	5.8–7.6	1.02	0.8–1.2	0.857
Pentoxifylline	190	6.5	5.6–7.4			
Other arrhythmias						
Cilostazol	89	3.1	2.4–3.7	1.14	0.8–1.5	0.392
Pentoxifylline	78	2.7	2.1–3.3			

*CI 95%* Confidence Interval with 95%, *HR* hazard ratio, *CAD* coronary artery disease, *AF* atrial fibrillation

^a^*p*-value computed using univariate Cox regression with robust standard errors by clusters (pairs)

Significant *p*-values are in boldface

We also conducted three subgroup analyses for the following groups of patients: 1) older than 65, 2) patients with type 2 DM, and 3) patients co-treated with antiplatelets. We did not find significant differences between the two groups of treatment in the subgroup of people older than 65 or in the patients with type 2 DM. For patients co-treated with antiplatelets, we found differences between cilostazol and pentoxifylline in the rates of other haemorrhages, which include eye bleeding, haemorrhages of the anus and rectum, epistaxis and haemorrhages not elsewhere classified; and in the rates of any haemorrhage. The incidence of these events was higher in pentoxifylline users than in cilostazol users (Table 3).

**Table 3:** Frequency and incidence of events in the population with co-treatment with antiplatelets (*n* = 3573)

		Risk at 5 years				
Outcomes	N	(%)	CI95%	HR	CI95%	*p* value^a^
Gastrointestinal haemorrhage						
Cilostazol	15	0.8	0.4–1.3	0.67	0.3–1.3	0.232
Pentoxifylline	22	1.2	0.7–1.8			
Cerebral haemorrhage						
Cilostazol	14	0.8	0.4–1.2	1.23	0.6–2.7	0.604
Pentoxifylline	13	0.7	0.3–1.1			
Other haemorrhages						
Cilostazol	55	3.1	2.3–3.9	0.65	0.5–0.9	**0.014**
Pentoxifylline	85	4.8	3.8–5.8			
Any haemorrhage						
Cilostazol	69	3.8	3.0–4.7	0.72	0.5–1.0	**0.040**
Pentoxifylline	98	5.5	4.5–6.6			
CAD						
Cilostazol	224	12.5	10.9–14.0	0.93	0.8–1.1	0.427
Pentoxifylline	237	13.3	11.8–14.9			
Stroke						
Cilostazol	103	5.7	4.7–6.8	0.83	0.6–1.1	0.180
Pentoxifylline	121	6.8	5.6–8.0			
AF						
Cilostazol	122	6.8	5.6–8.0	1.0	0.8–1.3	0.999
Pentoxifylline	120	6.8	5.6–7.9			
Other arrhythmias						
Cilostazol	61	3.4	2.6–4.2	1.42	1.0–2.1	0.082
Pentoxifylline	42	2.4	1.7–3.1			

*CI 95%* Confidence Interval with 95%, *HR* hazard ratio, *CAD* coronary artery disease, *AF* atrial fibrillation

^a^*p*-value computed using univariate Cox regression with robust standard errors by clusters (pairs)

Significant *p*-values in boldface

## Discussion

In this cohort study of the safety of cilostazol in PAD, we identified 9129 patients who initiated cilostazol or pentoxifylline during the study period. They differed in most of socio-demographic and clinical characteristics. After PS matching, the cohort was composed by 2905 cilostazol users and 2905 pentoxifylline users with comparable characteristics between groups.

We analysed the frequency and incidence of bleeding, cerebrovascular and cardiovascular events and we found non-statistically significant differences between cilostazol and pentoxifylline. We also conducted three sub-analyses in the following subgroups of patients: 1) population ≥ 65 years-old, 2) patients diagnosed with type 2 DM and 3) patients co-treated with antiplatelet agents. For patients co-treated with other antiplatelets, pentoxifylline showed higher incidences than cilostazol for overall haemorrhages, and for the group of other haemorrhages, which includes eye bleeding, haemorrhages of the anus and rectum, epistaxis and haemorrhages not elsewhere classified.

Our results are in line with previous studies, which did not find increases in haemorrhages, CAD or arrhythmic events in cilostazol-treated patients. In the clinical trial conducted by Dawson et al. [19] patients were randomized to receive cilostazol, pentoxifylline or placebo and the maximal walking distance was measured after 4, 8, 12, 16, 20 and 24 weeks. They also studied the frequency of common side effects and the group of “serious adverse events” had similar frequencies in the three groups, with non-significant differences.

In a systematic review of clinical trials comparing cilostazol with placebo or other drugs currently known to increase walking distance, such as pentoxifylline, 3718 patients were assessed and there was no clear evidence of a difference between any of the treatment groups and risk of AMI, stroke or all-cause mortality [20].

In the cohort study conducted by Leeper et al. [21], 232 patients with PAD treated with cilostazol were matched by 1:5 PS with 1160 patients not taking this drug. Over a mean follow-up of 4.2 years, they did not find association between cilostazol and cerebrovascular or cardiovascular events (OR for stroke 1.13, 95%CI 0.82–1.55; OR for AMI 1.00, 95%CI 0.71–1.39) or death (OR 0.86, 95%CI 0.63–1.18). They did not find increase in arrhythmic events either.

Previous published data in Spain [13] studied elderly patients with underlying comorbidities and receiving a considerable number of concomitant medications. The small sample size of the study does not allow establishing definite conclusions. Cilostazol should be used for IC only in patients with no contraindications for the treatment and should be used cautiously in elderly polymedicated patients, in those receiving two or more additional antiplatelet or anticoagulant agents and in those receiving strong inhibitors of CYP3A4 or CYP2C19.

### Strengths and limitations

The strengths of our study are the use of an automated health data which implies large number of patients included, representativeness for the general population, complete socio-demographic and health records, long follow-up, and real clinical practice data.

Some limitations of observational studies conducted with electronic health records are missing or incomplete information, prescriptions not linked with diagnoses coded and possible confounders. Specific limitations of our study are the possibly incomplete information on outcomes, as we only captured data of haemorrhages, CAD events or arrhythmias from the hospital discharges (CMBD-HA) and not from the records in PHC from SIDIAP. This may result in an infraestimation of events which could be related to cilostazol treatment. However, this would produce an infraestimation in both cohorts. Another limitation is the relatively low number of patients with PAD diagnosis coded during the study period, because ABPI determination was not implemented in all PHC centres in our setting. Nowadays, this problem is solved.

## Conclusion

Patients treated with cilostazol were clearly different from those treated with pentoxifylline at baseline, so they were matched through PS.

After matching, we did not find differences between treatment groups in the incidence of bleeding or cardiovascular and cerebrovascular events. We did not find differences in specific subgroups either, except an increase in overall haemorrhages rates with pentoxifylline in patients co-treated with additional antiplatelets.

Cilostazol should be used with precaution in elderly polymedicated patients in order to avoid adverse events.

## Additional file

Additional file 1: **Table S1**, which includes ICD9 and ICD10 diagnoses codes and ATC drug codes for the variables included in the study. (DOCX 17 kb)

## Abbreviations

ABPI: Ankle-brachial pressure index
ACEI: Angiotensin converter enzyme inhibitors
AMI: Acute myocardial infarction
ARB: Angiotensin receptor blocker
ATC: Anatomical therapeutic chemical classification
BMI: Body mass index
CAD: Coronary artery disease
CI: Confidence interval
CMBD-HA: Minimum set of data at hospital discharge
DM: Diabetes mellitus
ECAP: Electronic health records from primary healthcare
EMA: European medicines agency
HR: Hazard ratio
IC: Intermittent claudication
ICD: International classification of diseases
ICS: Institut català de la salut
LDL: Low-density lipoprotein
MDRD: Modification of diet in renal disease
NSAID: Non-steroidal anti-inflammatory drugs
OAC: Oral anticoagulants
OR: Odds ratio
PAD: Peripheral artery disease
PHC: Primary healthcare
PS: Propensity score
SIDIAP: Information system for research in primary care
SmPC: Summary of product characteristics
