# Supplementary material for: Safety of cilostazol in peripheral artery disease: a cohort from a primary healthcare electronic database
Source: BMC Cardiovasc Disord. 2018 May 8;18:85. doi: 10.1186/s12872-018-0822-4 (PMC5941464; doi:10.1186/s12872-018-0822-4)
Supplement: Supplementary file 1 — Table S1, which includes ICD9 and ICD10 diagnoses codes and ATC drug codes for the variables included in the study. (DOCX 17 kb) [file 12872_2018_822_MOESM1_ESM.docx]

## Additional file

### Table S1. ICD9 and ICD10 diagnosis codes and ATC drug codes.

| **Variables** | | | |
| --- | --- | --- | --- |
| **Diagnosis** | **ICD10 codes (PHC)** | **ICD9 codes (hospital)** | |
| Peripheral artery disease | I70.2, I73.8, I73.9 |  | |
| Hypertension | I10-I15 |  |  |
| Type 2 diabetes mellitus | E11-E14 |  |  |
| Dyslipidemia | E78 |  |  |
| Stroke | G45.0, G45.8, G45.9, G46.4, G46.5, G46.6, G46.7, G46.8, I63, I64, I69 | 435.0, 435.3, 435.9, 437.8, 434.01, 434.11, 434.91 | |
| Coronary artery disease | I20-I24 | 410, 411, 412, 413, 414 | |
| Arrhythmia  Atrial fibrillation  Other arrhythmias | I48  I45, I47, I49 | 427.3  426, 427 | |
| Haemorrhages overall  Cerebral  Gastrointestinal      Other haemorrhages | I60-I62, S06.4  K22.8, K25.0, K25.2, K25.4, K25.6, K26.0, K26.2, K26.4, K26.6, K27.0, K27.2, K27.4, K27.6, K28.0, K28.2, K28.4, K28.6, K29.0, K92.0, K92.1, K92.2  H11.3, H35.6, K62.5, R04.0, R58 | 430, 431, 432  531.0, 531.2, 531.4, 531.6, 533.0, 533.2, 535.01  534.0, 534.2, 530.82 | |
| **Drug exposure** | **ATC code** | |  |
| Cilostazol | B01AC23 | |  |
| Pentoxifylline | C04AD03 | |  |
| Diuretics | C03 | |  |
| β-blockers | C07 | |  |
| Calcium channel antagonists | C08CA, C08DA, C08DB | |  |
| ACEI | C09AA, C09BA, C09BB | |  |
| ARB | C09CA, C09DA, C09DB, C09DX | |  |
| Lipid modifying agents | C10A | |  |
| Antidiabetics and insulins | A10A, A10B | |  |
| OAC | B01AA, B01AB, B01AE07, B01AF, B01AX06 | |  |
| Antiplatelets | B01AC04, B01AC05, B01AC06, B01AC07, B01AC22, B01AC24 | |  |
| NSAID | M01AB, M01AC, M01AE, M01AH, N02BA, N02BB | |  |
| Proton pump inhibitors | A02BC | |  |
| Nitrates and other vasodilators | C01DA, C01EB15, C01EB17, C01EB18, C01DX12, C02DB02 | |  |

*ACEI, angiotensin-converter enzyme inhibitors; ARB, angiotensin-receptor blockers; OAC, oral anticoagulants; NSAID, non-steroidal anti-inflammatory drugs
